# Improving sport-based psychosocial interventions in Europe: results from the EASMH training programme for professional sport coaches

**DOI:** 10.1192/j.eurpsy.2023.694

**Published:** 2023-07-19

**Authors:** G. Sampogna, M. Di Vincenzo, M. Borgi, B. Collacchi, F. Cirulli, S. Cerino, S. Rullo, M. Luciano, V. Di Tommaso, S. Moliterni, A. Bichi, J. Garside, S. Kivistö, A. Iarion, A. Fiorillo

**Affiliations:** ^1^University of Campania, Naples; ^2^ National Institute of Health; ^3^ECOS, Rome, Italy; ^4^EPSI, Brussels, Belgium; ^5^Everton in the Community, Everton, United Kingdom; ^6^Finnish Sport Federation Tempere, Tempere, Finland; ^7^University of Costanta, Costanta, Romania; ^8^University of Campania, EPA Board Member, Naples, Italy

## Abstract

**Introduction:**

In the framework of the EU-Erasmus+, the European Alliance for Sport and Mental Health (EASMH) project has been funded, aiming to promote the improvement of good clinical practice for sport-based psychosocial interventions throughout Europe. A specific training programme tailoring professional sport coaches has been developed in order to improve their skills in engaging and involving patients with severe mental disorders in sport-based rehabilitation activities.

**Objectives:**

to evaluate the perceived quality and utility of the EASMH training programme by sport coaches from different European countries (including Italy, UK, Romania, and Finland).

**Methods:**

As part of the EASMH project, the University of Campania “L. Vanvitelli” has coordinated the development of training materials for professional sport coaches. The training programme has been tested in a pilot training programme. An ad-hoc questionnaire has been developed and administered at the end of the training, during a meeting held in Brussels in July 2022.

**Results:**

The EASMH training programme consists of six modules, dealing with the following topics: definition of mental health/mental disorders; classification systems; essential clinical features of severe mental disorders; personal and social burden associated with severe mental disorders; how to build a therapeutic relationship with a patient with severe mental disorders; verbal and non-verbal communication; evaluation of patient’s preference in selecting sport activities; definition of a personalized plan; motivational interview/problem-solving strategy. A total of eight professional coaches involved in different sport coming from Italy, Romania, United Kingdom and Finland participated in the entire training, consisting of six 4hr training modules. Seven out the eight coaches compiled the questionnaire. The overall feedback has been extremely positive. Overall, coaches have judged the modules as very clear, useful and of high standing. Each question has been rated with an average of 4.35 related to the overall content.

**Conclusions:**

The present survey confirms that a short online training programme focused on professional sport coaches is well received by participants and can provide them with useful information on how to engage patients with severe mental disorders. The next step of the EASMH project foresees the implementation of several local pilot actions with the active involvement of patients with severe mental disorders.

**Disclosure of Interest:**

None Declared

